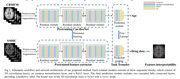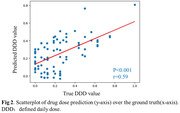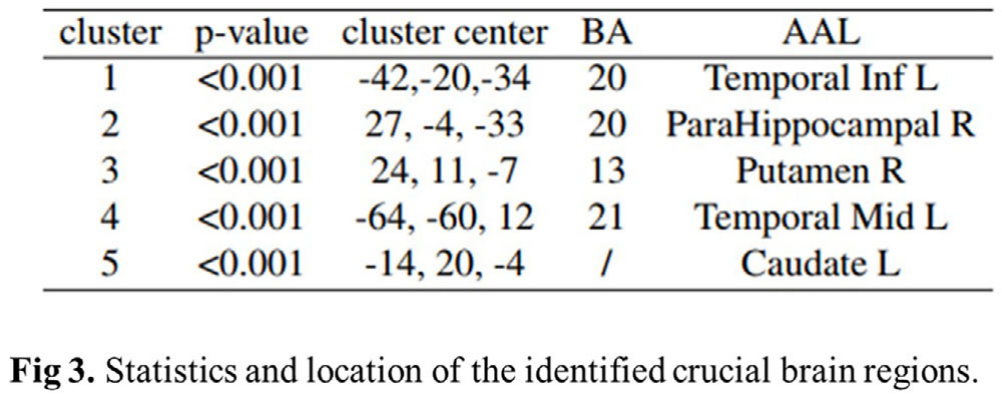# Predicting Antipsychotic Drug Doses for BPSD: A Transfer Learning Approach Using Neuroimaging Data

**DOI:** 10.1002/alz.093748

**Published:** 2025-01-09

**Authors:** Ling Yue, Bo Hong, Tianli Tao, Xia Li, Shifu Xiao, Han Zhang

**Affiliations:** ^1^ Alzheimer's Disease and Related Disorders Center, Shanghai Jiao Tong University, Shanghai China; ^2^ Shanghai Mental Health Center, Shanghai Jiao Tong University School of Medicine, Shanghai China; ^3^ School of Biomedical Engineering, ShanghaiTech University, Shanghai China

## Abstract

**Background:**

About 50‐90% people with dementia would develop behavioral disturbances, namely, behavioral and psychological symptoms of dementia (BPSD). Antipsychotic medications are widely used to control severe BPSD symptoms which suffers serious safety risks. It is challenge for individualized precise prediction of antipsychotic drug doses. Neuroimaging, particularly MRI, reveals brain structure associated with aging, cognitive decline, and psychiatric symptoms, making it a potential tool for predicting the drug doses. This study employs transfer learning to predict drug dose and offer neuroanatomical interpretation of BPSD from the perspective of deep learning.

**Method:**

We employed a two‐step process to train our model (Figure 1). Initially, a large dataset from the Chinese Brain Molecular and Functional Mapping (CBMFM) project (n=646,334 females and 312males, age 18‐82) was used to pretrain the model with a brain age prediction task. Subsequently, the pretrained model was fine‐tuned for drug dose prediction from the Alzheimer's Disease and Related Disorders Center in Shanghai Jiao Tong University (ADRDC) dataset (83 BPSD patients,27 males and 56 females, age 55‐80). Finally, we utilized gradient‐weighted class activation mapping to generate attention maps and conducted statistical analyses on the attention maps to identify critical brain regions for drug dose prediction. To determine the individual usage of different antipsychotic drugs, the concept of defined daily dose (DDD) was used. The DDD, which individualized control the BPSD, was calculated, ranging from 0 to 1.5 mg/day, serving as the label for fine‐tuning.

**Result:**

Our pretrained Cas‐ResNet exhibited enhanced performance with fewer training epochs, achieving a competitive Pearson correlation of 0.59 between estimated and real DDD (Figure 2). Through feature interpretability analysis, we identified five significant brain regions crucial for BPSD drug dose prediction, mainly located in the temporal lobe, including the parahippocampal area and the striatum (putamen and caudate)(Figure 3). These findings indicate that the antipsychotic dosage to control the BPSD is linked to brain structural alterations, involving both dementia‐related and emotion‐regulating areas.

**Conclusion:**

For the first time, we showed a promising result of using a lightweight deep learning model to predict drug dose prescribed for controlling BPSD. The work promotes the discussion toward appropriate use of antipsychotics in patients with dementia.